# Assessment of phytochemicals, antioxidant, anti-lipid peroxidation and anti-hemolytic activity of extract and various fractions of *Maytenus royleanus* leaves

**DOI:** 10.1186/1472-6882-13-143

**Published:** 2013-06-22

**Authors:** Maria Shabbir, Muhammad Rashid Khan, Naima Saeed

**Affiliations:** 1 Department of Biochemistry, Faculty of Biological Sciences, Quaid-i-Azam University Islamabad, Islamabad 45320, Pakistan

**Keywords:** *Maytenus Royleanus*, Antioxidant Activities, Phenolic Content, Solvent Extraction

## Abstract

**Background:**

*Maytenus royleanus* is traditionally used in gastro-intestinal disorders. The aim of this study was to evaluate the methanol extract of leaves and its derived fractions for various antioxidant assays and for its potential against lipid peroxidation and hemolytic activity.

**Methods:**

Various parameters including scavenging of free-radicals (DPPH, ABTS, hydroxyl and superoxide radical), hydrogen peroxide scavenging, Fe^3+^ to Fe^2+^ reducing capacity, total antioxidant capacity, anti-lipid peroxidation and anti-hemolytic activity were investigated. Methanol extract and its derived fractions were also subjected for chemical constituents. LC-MS was also performed on the methanol extract.

**Results:**

Qualitative analysis of methanol extract exhibited the presence of alkaloids, anthraquinones, cardiac glycosides, coumarins, flavonoids, saponins, phlobatannins, tannins and terpenoids. LC-MS chromatogram indicated the composition of diverse compounds including flavonoids, phenolics and phytoestrogens. Methanol extract, its ethyl acetate and *n*-butanol fractions constituted the highest amount of total phenolic and flavonoid contents and showed a strong correlation coefficient with the IC_50_ values for the scavenging of DPPH, hydrogen peroxide radicals, superoxide radicals, anti-lipid peroxidation and anti-hemolytic efficacy. Moreover, *n*-butanol fraction showed the highest scavenging activity for ABTS radicals and for reduction of Fe^3+^ to Fe^2+^.

**Conclusions:**

Present results suggested the therapeutic potential of *Maytenus royleanus* leaves, in particular, methanol extract, ethyl acetate and *n*-butanol fraction as therapeutic agent against free-radical associated damages. The protective potential of the extract and or fraction may be attributed due to the high concentration of phenolic, flavonoid, tannins and terpenoids.

## Background

Nowadays, plants provide raw materials for new sources of drugs and pharmaceutical products. A wide variety of naturally occurring constituents such as polyphenolics, terpenoids and pro-vitamins have received much attention as alternative therapeutic agents to fight against various oxidative stress induced diseases [1-3]. Numerous natural antioxidants from medicinal plants have been isolated and characterized. In particular, phenolic compounds are suggested to exert therapeutic activities because of their anti-oxidative and anti-inflammatory properties. The polyphenolic compounds impart multiple antioxidant properties such as scavenging of free radicals, reducing abilities or act as metal chelators. Furthermore, anti-microbial, anti-thrombotic and vasodilator properties of phytochemicals have increased the interest as alternative and complementary medicines [4]. The ability of cells to detoxify the reactive intermediates that are produced as a result of excessive metabolism is compromised, which disturbs the equilibrium between antioxidant and reactive oxygen species (superoxide radical, hydroxyl radical, peroxide radical, and nitric oxide). In addition to the threats posed by oxidative stress to biological system, it is also responsible for inducing chronic diseases (cancer, cardiovascular, aging, diabetes, cataract) [5]. Various studies have suggested an association between the dietary intake of these phytochemicals and the prevention of various stress induced anomalies [6-11]. It is need of the day that medicinal plants should be characterized for their pharmacological properties as majority of the population in developing countries use traditional medicines [11-13].

Antioxidants (polyphenols, vitamin C, vitamin E, selenium, β-carotene, lycopene, lutein and other carotenoids) present in large amounts in many medicinal plants and herbs, eliminate free radicals by acting as antioxidant, by neutralizing, quenching, reducing or through decomposing peroxides [14]. Compounds carrying antioxidant potential can be isolated and used as a remedy against oxidative stress and related diseases [15]. Recent studies are focusing on replacement of synthetic antioxidants with naturally occurring antioxidants to avoid the potential toxicity of synthetic ones [16-18].

*Maytenus royleanus* belongs to the family Celastraceae, Its flowering season is March-April and is distributed throughout lower Kaghan on dry sunny slopes. It is a shrub having stiff branches, usually with straight thorns, white flowers in short axillary clusters, fruit is 3 angled capsule [19]. Paste of bark is applied with mustard oil to destroy pediculi and it is also used locally against gastro-intestinal diseases [20]. Smoke of the seeds is believed to be valuable against toothache. Leaves are often used as fodder. Branches are used for repair of houses and fuel. In spite of the popular use, there is no report about phytochemical composition and antioxidant properties of *M. royleanus* leaves. Involvement of oxidative stress as a key role in various diseases, we aimed to evaluate the phytochemical and antioxidant potential of this plant.

## Methods

### Chemicals

All the chemicals used in these assays were of high quality. Na_2_CO_3,_ ascorbic acid, gallic acid, rutin, Folin-Ciocalteu’s phenol reagent, NaNO_2_, AlCl_3_.6H_2_O, rutin, 2, 2-Diphenyl-1-Picrylhydrazyl (DPPH), 2,2- azino-bis (3-ethylbanzthiazoline-6- sulphonic acid (ABTS), potassium oxidopersulphate, H_2_SO_4,_ ammonium molybdate, riboflavin, phenazine methosulphate (PMS), nitroblue tetrazolium (NBT), H_2_O_2,_ 2- deoxyribose, ferric chloride, potassium chloride, trichloroacetic acid (TCA), thiobarbituric acid (TBA), potassium ferricynide, Mayer’s reagent, NaOH, FeCl_3_ were obtained from Sigma Chemicals Co. (St. Louis, MO, USA). Solvents and other reagents were of analytical grade.

### Collection of plant

Collection of the plant material was made in March 2011 from village Lehtrar, Tehsil Kotli Sattian of Pakistan. After identification a voucher specimen (# 032564) was deposited at the Herbarium of Pakistan Museum of Natural History, Islamabad.

### Preparation of extract

Powder of dried leaves (500 g) was extracted with 95% methanol twice (2000 ml for each) for 48 h with occasional shaking and filtered. Extract was dried at 40°C under reduced pressure giving a yield of 12.2% to that of the powder (Panchun Scientific Co., Kaohsiung, Taiwan). Extract was suspended in 50 ml of distilled water and fractions were made by adding solvents (200 ml twice) successively with increasing polarity i.e., *n*-hexane, chloroform, ethyl acetate, *n*-butanol and shake vigorously. The layers were separated accordingly and the soluble remainder was used as residual aqueous fraction. Fractions were dried with the yields *n*-hexane (7.5%), chloroform (3.4%), ethyl acetate (8.9%), *n*- butanol (7.8%) and residual aqueous fraction (10.7%) to that of the methanol extract [21].

### Phytochemical screening

The methanol extract of *M. royleanus* was analyzed by LC-MS to get the fingerprint of compounds it carries and was found to carry number of compounds (retention time: 0.5-45 min, m/z “mass” restriction: 100-3200 Da, peak height > 500 counts, relative height: > 2.5%, limit to largest: 300 compounds). Dried sample of methanol extract (~15 mg), was dissolved 1 ml of methanol (15 mg/ml). It was spun down to remove particles and 10 μl was injected under C18 or HILIC chromatography and positive (C18) or negative (C18/HILIC) ionization (targeting flavonoids and phenolic acids).

### Qualitative determination of the chemical constituents

Presence of alkaloids, anthraquinones, cardiac glycosides, coumarins, flavonoids, saponins, phlobatannins, tannins and terpenoids in the extract and various fractions was confirmed individually by following standard procedures.

#### Test for alkaloids

Mixture of methanol extract of *M. royleanus* leaves and its various derived fractions (0.4 g) in 8 ml of 1% HCl was warmed on water bath. After filtration 2 ml filtrate from the extract and each fraction was allowed to react with few drops of potassium mercuric iodide and with potassium bismuth, separately. Turbidity or precipitation formation was considered as a confirmation for presence of alkaloids [22].

#### Test for saponins

The criterion of oil emulsion formation of saponins was used for the screening of saponins [22]. Briefly, extract and various fractions (20 mg) suspended in 20 ml of distilled water and boiled for 5 min. In 10 ml of the above filtrate 5 ml of distilled water was added and mixed well to develop the froth. Development of emulsion after mixing the froth with olive oil confirmed the existence of saponins.

#### Test for terpenoids

Briefly, 2 ml of chloroform was mixed with 5 ml (1 mg/ml) of each sample in a test tube then 3 ml of concentrated H_2_SO_4_ was added to develop the color. Exhibition of reddish brown coloration at the interface confirmed the presence of terpenoids [22].

#### Test for anthraquinones

To a volume of 6 ml of 1% HCl, 200 mg of each sample was added separately and boiled. Benzene (5 ml) was mixed with the filtrate and after separation of benzene layer 2 ml of 10% ammonia solution was lowered. Development of pink, violet or red color in the ammonical phase indicated the existence of anthraquinones [22].

#### Cardiac glycosides determination

An aliquot of 5 ml of methanol extract of *M. royleanus* leaves and its various fractions (10 mg/ml in methanol) were added in the sequence of glacial acetic acid (2 ml) and FeCl_3_ solution (one drop). Concentrated H_2_SO_4_ (1 ml) was added and the formation of brown ring at the interface confirmed the presence of cardiac glycosides [23].

#### Test for coumarins

In a vial having 300 mg/ml of the extract and each fraction was plugged with filter paper dipped in 1 N NaOH and boiled in a boiling water bath for few minutes. Yellow fluorescence of filter paper under UV light confirmed the presence of coumarins [23].

#### Test for phlobatannins

An amount of 80 mg of the extract and various fractions was boiled in 1% HCl. Development of red precipitate indicated the existence of phlobatannins [23].

#### Test for flavonoids

Mixture of methanol extract and various fractions of *M. royleanus* leaves were prepared by adding 50 mg of each sample to 100 ml of distilled water and filtered. An aliquot of 5 ml of dilute ammonia solution was mixed with 10 ml of the filtrate. Appearance of yellow coloration by addition of few drops of concentrated sulfuric acid indicated the presence of flavonoid [24].

#### Test for tannins

A mixture was prepared by mixing 50 mg of methanol extract and each fraction in 20 ml of distilled water and boiled. Appearance of brownish green or blue-black coloration after mixing few drops of 0.1% FeCl_3_ confirmed the existence of tannins [24].

### Quantitative determination of the chemical constituents

#### Quantification of alkaloids

Quantification of alkaloids was performed by following the method reported previously (22). Each fraction (50 mg) was mixed with 200 ml of acetic acid (10%) in ethanol; the beaker was covered and incubated for 4 h. The mixture was concentrated up to one third of its total volume. Ammonium hydroxide was added drop wise in the mixture until it formed precipitate. The precipitate was washed with ammonium hydroxide and then filtered. The filtrate (alkaloids) was calculated as percentage of the dried fraction.

#### Quantification of tannins

For quantification of tannins each fraction (50 mg) was suspended in 100 ml of distilled water, it was shaken for 1 h in a mechanical shaker and then filtered. Each sample (5 ml) was added with ferric chloride (2 ml) in hydrochloric acid (0.1 N) and potassium ferricyanide (0.008 M). Absorbance was taken at 120 nm within 10 min and tannins contents were calculated as percentage of the dried fraction (24).

#### Quantification of saponins

Methanol extract and each fraction (50 mg) were mixed in 100 ml of ethanol (20%). It was kept on heating for 4 h with continuous stirring at 55°C, than diluted with diethyl ether (20 ml) and washed with 5% sodium chloride. Saponins were estimated as percentage of the dried fraction (22).

#### Estimation of leaf protein

For the determination of leaf protein content, monobasic sodium phosphate (16 ml) and dibasic sodium phosphate (84 ml) was combined to get the desire pH (7.5) of phosphate buffer.

Reagent 1: Sodium carbonate (2 g), sodium chloride (0.4 g) 0.1 N and Na-K tartarate 1 g was dissolved in distilled water (100 ml).

Reagent 2: Copper sulphate 0.5 g was mixed in distilled water (100 ml).

Reagent 3: Solution A (50 ml) and solution B (1 ml) both were mixed in a flask.

Reagent 4: Folin phenol reagent was added to distilled water (1:1 ratio).

Fresh leaves (0.1 g) were homogenized in 1 ml of phosphate buffer (pH 7.5). The homogenate was centrifuged for 10 min at 3000 rpm. The 0.1 ml of supernatant was taken in a test tube and finally volume was raised by adding distilled water up to 1 ml. Reagent D (0.1 ml) was mixed after shaking for 10 min. After 30 min of incubation, absorbance of each sample was taken at 650 nm and protein content was measured with help of standard (BSA) (23).

#### Sugar estimation

Fresh plant material was used to estimate the sugar content; plant material was homogenized and then treated with concentrated sulphuric acid. The sample was incubated for 4 h at 25°C. Optical density of each sample was observed at 420 nm (24).

#### Total phenolic content

Briefly, a mixture was prepared by adding 9 ml of distilled water and 1 ml of each sample. To the mixture 1 ml of Folin-Ciocalteu’s phenol reagent was added followed by the addition of 10 ml of Na_2_CO_3_ solution and total volume was made to 25 ml by adding distilled water. The optical density was determined after 90 min at 750 nm at 23°C. Quantity of total phenolics was determined as mg of gallic acid equivalent (GAE) per g of dried sample. For blank distilled water was used [25].

#### Total flavonoid content

According to this method [26], 0.3 ml of the filtrate the extract and each fraction (50 mg) in 10 ml of 80% methanol was mixed with a reagent; 3.4 ml of 30% methanol, 0.15 ml of NaNO_2_ (0.5 M) and 0.15 ml of AlCl_3_.6H_2_O (0.3 M). Then NaOH was added properly mixed. Absorbance was recorded at 506 nm after 5 min of mixing. Total flavonoid content was calculated as mg of rutin equivalent per g of dried extract or fraction.

#### *In vitro* antioxidant assays

A stock solution (1 mg/ml) of the methanol extract and each fraction was prepared in 95% methanol and diluted accordingly for various antioxidant and reducing assays. Antioxidant power of each assay was compared with the efficacy of standard chemicals.

#### DPPH radical scavenging activity

Antioxidant potential of the extract and each fraction was assessed by using 1,1-diphenyl 1-2-picryl-hydrazyl (DPPH) assay [27]. DPPH (2.4 mg) was dissolved in 100 ml of methanol and diluted with methanol to obtain an absorbance of about 0.98 (± 0.02) at 517 nm. An aliquot of 0.01 ml of the extract and each fraction at different concentrations of 25-250 μg/ml was added in 3 ml of the DPPH solution. After incubation for 15 min in dark; absorbance of the mixture was determined at 517 nm. Following formula was applied to determine the DPPH radical scavenging activity;

$$\begin{array}{rl} \mathrm{Percentage}\;\mathrm{inhibition}= & \left[\left(\mathrm{control}\;\mathrm{absorbance}-\mathrm{sample}\;\mathrm{absorbance}\right)\right)\\ & \left(/\left(\mathrm{control}\;\mathrm{absorbance}\right)\right]\times 100. \end{array}$$

IC_50_ is the concentration value which scavenged 50% of the DPPH radicals. Ascorbic acid and rutin were used as reference compounds [28,29].

#### Superoxide radical scavenging

Procedure of Beauchamp and Fridovich [30] was followed to determine the scavenging potential of the extract and fractions with respect to superoxide radicals. Briefly, 0.5 ml of 50 mM phosphate buffer (pH 7.6), 0.3 ml of 50 mM riboflavin, 0.25 ml of 20 mM phenazine methosulphate (PMS) and 0.1 ml of 0.5 mM nitro blue tetrazolium (NBT) were mixed, before the addition of 100 μl of the methanol extract and each fraction at varying concentrations of 25-250 μg/ml. Mixture was illuminated in the fluorescent light for 20 min and 560 nm wave length was used to record the absorbance of the mixture. The percent inhibition was calculated by using the following formula:

$$\begin{array}{rl} \mathrm{Percentage}\;\mathrm{inhibition}= & \left(1-\mathrm{absorbance}\;\mathrm{of}\;\mathrm{sample}\right)\\ & \left(/\mathrm{absorbance}\;\mathrm{of}\;\mathrm{control}\right)\times 100. \end{array}$$

Reference compound used was ascorbic acid in this assay.

#### Total antioxidant capacity

Phosphomolybdate assay system was used to determine the total antioxidant activity of the methanol extract and various fractions [31]. To a reagent solution; sulphuric acid (0.6 M), sodium phosphate (28 mM) and ammonium molybdate (4 mM); 100 μl of each sample was added and incubated at 95°C in a water bath for 90 min. After cooling to room temperature; absorbance was recorded at 765 nm against reagent blank. Total antioxidant capacity of the ascorbic acid was also estimated for reference. The total antioxidant capacity was determined by using following formula:

$$\begin{array}{l} \mathrm{Total}\;\mathrm{Antioxidant}\;\mathrm{capacity}\left(\%\right)\\ \;=\left[\left(\mathrm{control}\;\mathrm{absorbance}-\mathrm{sample}\;\mathrm{absorbance}\right)\right)\\ \;\left(/\left(\mathrm{control}\;\mathrm{absorbance}\right)\right]\times 100. \end{array}$$

### Reducing assays

#### Hydroxyl radical scavenging

A method described earlier was adapted to assess the hydroxyl radical scavenging ability of various samples [32]. Briefly, reagent solution was prepared by sequential addition of ferric chloride (10 mM), 0.25 ml of 2-deoxyribose (2.8 mM) in 50 mM phosphate buffer (pH 7.4), 0.1 ml of 1 mM (1:1; v/v) EDTA solution and 0.1 ml of 10 mM H_2_O_2_. A volume of 0.1 ml of the extract and various fractions was individually added to 0.01 ml of reagent solution. Then 0.1 ml of ascorbate (1 mM) was added and incubated at 37°C for 1 h. In the mixture thiobarbituric acid (TBA) 0.5%; w/v in 1 ml of 50 mM NaOH and 1 ml of 10% w/v trichloroacetic acid (TCA) was added and cooled to room temperature after incubation in a boiling water bath for 15 min. Intensity of chromogen was read at 532 nm. The hydroxyl radical scavenging activity was estimated as;

$$\begin{array}{l} \mathrm{Hydroxyl}\;\mathrm{radical}\;\mathrm{scavenging}\;\mathrm{activity}\left(\%\right)\\ \;=\left(1-\mathrm{Abs}.\mathrm{of}\;\mathrm{sample}/\mathrm{Abs}.\mathrm{of}\;\mathrm{control}\right)\times 100. \end{array}$$

#### Hydrogen peroxide scavenging

Methods described previously [33-35] were followed to determine the ability of extract and various fractions to scavenge H_2_O_2_. Hydrogen peroxide (2 mM) working solution was made by mixing with 50 mM phosphate buffer (pH 7.4). Reaction mixture was prepared by the addition of 0.1 ml of extract and each fraction with 0.4 ml of 50 mM phosphate buffer (pH 7.4) followed by the addition of 0.6 ml of 50 mM H_2_O_2_ and allowed to stand for 10 min. At 230 nm absorbance of the mixture was recorded. Following equation was used to determine the capacity to scavenge H_2_O_2_;

$$\begin{array}{l} \mathrm{Hydrogen}\;\mathrm{peroxide}\;\mathrm{scavenging}\;\mathrm{activity}\left(\%\right)\\ \;=\left(1-\mathrm{absorbance}\;\mathrm{of}\;\mathrm{sample}/\mathrm{absorbance}\;\mathrm{of}\;\mathrm{control}\right)\\ \;\times 100 \end{array}$$

#### ABTS radical scavenging

Method of Re et al. [36] was used for the ABTS radical scavenging activity of the extract and each fraction. For the development of ABTS radicals potassium persulfate (2.45 mM) solution was mixed with ABTS (7 nM) and incubated overnight in the dark to get a dark colored solution. The standard solution of ABTS was diluted by the addition of 60% methanol to have an absorbance of 0.70 (± 0.02) at 745 nm at 30°C. An aliquot of 0.3 ml of extract or fraction was mixed with 1 ml of ABTS and absorbance was recorded after one minute. Reducing capacity was determined according to the formula:

$$\begin{array}{rl} \%\;\mathrm{inhibition}= & \left[\left(\mathrm{control}\;\mathrm{absorbance}-\mathrm{sample}\;\mathrm{absorbance}\right)\right)\\ & \left(/\left(\mathrm{control}\;\mathrm{absorbance}\right)\right]\times 100. \end{array}$$

#### Reduction of Fe^3+^ to Fe^2+^

Reducing capability of the extract and each fraction was estimated by following the method of Oyaizu [37]. A volume of 2 ml of 0.2 M phosphate buffer (pH 6.6) and 2 ml of potassium ferricyanide was mixed with 2 ml of the extract and each fraction (10 mg/ml) and incubated for at 50°C for 20 min. From the reaction mixture 2 ml was taken after the addition of 2 ml of 10% TCA and was mixed with 0.4 ml of 0.1% ferric chloride and 2 ml of distilled water. After 10 min of incubation optical density of the chromogen formed was read at 700 nm. High reducing power ability was associated with high absorbance values. Reducing power of ascorbic acid was considered as reference.

#### *In vitro* anti-lipid peroxidation assay

Standard method for estimation of TBARS was used to assay the degree of lipid peroxidation [38]. The study procedure for the animal care and experimentation was permitted by Ethical Committee of Quaid-i-Azam University Islamabad. From freshly excised liver of rat 10× homogenate was made in cold phosphate buffer saline (pH 7.4). Extract and each fraction were added to 100 μl of (15 mM) ferrous sulphate followed by addition of 3 ml of homogenate. After incubation for 30 min; 0.1 ml of this reaction mixture was mixed with 1.5 ml of 10% TCA. After 10 min of incubation it was filtered and supernatant was added in a tube having 1.5 ml of 0.67% TBA (in 50% acetic acid) and placed in a boiling water bath for 30 min. Concentration of chromogen formed was measured at 535 nm. Anti-lipid peroxidation was assessed by using the following formula:

$$\%\;\mathrm{Inhibition}=\left(\mathrm{control}-\mathrm{test}\right)/\mathrm{control}\times 100$$

### Anti-hemolytic activity

Anti-hemolytic activity was assessed by following the spectrophotometric method [39]. From a normal healthy individual 5 ml of blood was taken and centrifuged at 1500 rpm for 3 min. Pellet of blood was washed three times in sterile phosphate buffer saline solution (pH 7.2). The pellet was re-suspended in normal 0.5% saline solution. A volume of 0.5 ml of the extract and various fractions (10, 50, 100, 200, 250 μg/ml in saline) were added in 0.5 ml of cell suspension. After incubation the mixture at 37°C for 30 min it was centrifuged at 1500 rpm for 10 min. Anti-hemolytic activity was assessed by measuring the absorbance at 540 nm. For positive and negative control distilled water and phosphate buffer saline were used respectively. The study protocol was in compliance with Helsinki Declaration.

### Statistical analysis

*In vitro* and other parametric assays were performed in triplicate and results are shown as mean ± SD. Antioxidant potential of different assays was determined as IC_50_ values by applying Graph pad prism 5-software. Statistical significance was determined among various treatments with one way ANOVA test. A statistical significance of P < 0.05 or P < 0.01 was considered to be significant.

## Results

### Phytochemical analysis

Phytochemical analysis of methanol extract of *M. royleanus* leaves indicated the existence of alkaloids, anthraquinones, cardiac glycosides, coumarins, flavonoids, saponins, phlobatannins, tannins and terpenoids. Saponins, phlobatannins and terpenoids were not determined in *n*-hexane fraction while alkaloids, coumarins, saponins, phlobatannins and terpenoids were not detected in chloroform fraction. Anthraquinones and cardiac glycosides were not detected in ethyl acetate and in *n*-butanol fraction respectively. Residual aqueous fraction depicted the existence of anthraquinones, coumarins, phlobatannins and tannins (Tables 1 and 2).

**Table 1:** **Estimation of phytochemicals of extract and its fractions of *M. royleanus *leaves**

**Phytochemical tests**	**Methanol**	***n*-hexane**	**Chloroform**	**Ethyl acetate**	***n*-butanol**	**Residual aqueous**
	**extract**	**fraction**	**fraction**	**fraction**	**fraction**	**fraction**
Alkaloids	+	+	-	+	+	-
Anthraquinones	+	+	+	-	+	+
Cardiac glycosides glycosides	+	+	+	+	-	-
Coumarins	+	+	-	+	+	+
Flavonoids	+	+	+	+	+	-
Saponins	+	-	-	+	+	-
Phlobatannins	+	-	-	+	+	+
Tannins	+	+	+	+	+	+
Terpenoids	+	-	-	+	+	-

+, present; -, absent.

**Table 2:** **Quantitative estimation of phytochemicals of extract and its fractions of *M. royleanus *leaves**

**Samples**	**Saponins%**	**Tannins%**	**Alkaloids%**	**Protein (mg/g weight)**	**Sugar (mg/g weight)**
Methanol extract	7.05 ± 0.07	5.57 ± 0.02	1.84 ± 0.02	6.65 ± 0.45	2.03 ± 0.08
*n*-hexane fraction	6.4 ± 0.2	3.28 ± 0.12	0.74 ± 0.12	4.21 ± 0.01	1.01 ± 0.03
Chloroform fraction	1.76 ± 0.09	5.27 ± 0.14	1.14 ± 0.14	0.65 ± 0.11	0.01 ± 0.4
Ethyl acetate fraction	5.16 ± 0.01	4.89 ± 0.2	0.54 ± 0.2	4.56 ± 0.03	1.22 ± 0.02
*n*-butanol fraction	5.82 ± 0.04	3.12 ± 0.09	0.63 ± 0.09	2.23 ± 0.05	1.09 ± 0.21
Residual aqueous fraction	3.08 ± 0.11	4.12 ± 0.08	0.05 ± 0.08	1.21 ± 0.04	1.23 ± 0.04

Each value is represented as mean ± SD (n = 3).

### Extraction yield

The percentage extraction yield of methanol extract and its derived fractions is shown in Table 2. The extraction yield of methanol extract was 12.2 ± 0.9% to that of the dry powder. Extraction yield of different fractions was obtained in descending order of residual aqueous > ethyl acetate > *n*-butanol > *n*-hexane > chloroform fraction. There was found a significant (P < 0.05) difference for the extraction yield for all the fractions except nonsignificant (P > 0.05) difference was recorded for the extraction yield of *n*-hexane and *n*-butanol fraction.

### Estimation of total flavonoid and phenolic content

Methanol extract showed the total phenolic content of 76.0 ± 2.7 mg gallic acid equivalent/g dried powder. Total phenolic content of various fractions was determined in the descending order of ethyl acetate > *n*-butanol > *n*-hexane > residual aqueous > chloroform fraction having significant (P < 0.05) differences among themselves (Table 3). In case of total flavonoid content estimation, methanol extract exhibited the total flavonoid content of 63.5 ± 1.84 mg rutin equivalent/g dried fraction. Significant (P < 0.05) difference was recorded in the descending order for; ethyl acetate > *n*-butanol > *n*-hexane > residual aqueous > chloroform fraction.

**Table 3:** **Estimation of total phenolic, flavonoid and extraction yield of extract and fractions of *M. royleanus *leaves**

**Plant extract**	**Total phenolics**	**Total flavonoid**	**Extraction yield (%)**
	**(mg GAE/g of extract)**	**(mg rutin equivalent/g of extract)**	
Methanol extract	76 ± 2.7^a^	63.5 ± 1.84^a^	12.2 ± 0.9^a^
*n*-hexane fraction	44.5 ± 3.23^d^	36.8 ± 1.94^d^	7.5 ± 1.45^d^
Chloroform fraction	6.8 ± 3.93^f^	9.3 ± 1.76^e^	3.4 ± 0.28^e^
Ethyl acetate fraction	62.3 ± 2.77^b^	60.9 ± 1.83^b^	8.9 ± 1.11^c^
*n*-butanol fraction	53 ± 2.63^c^	52.5 ± 1.13^c^	7.8 ± 0.43^d^
Residual aqueous fraction	28.4 ± 3.71^e^	31.4 ± 1.27^d^	10.7 ± 0.5^b^

Each value is represented as mean ± SD (n = 3). Values in the same column followed by a different letter (^a-f^) are significantly different (P < 0.05).

### Antioxidant assays

#### DPPH radical scavenging activity

**Figure 1:**
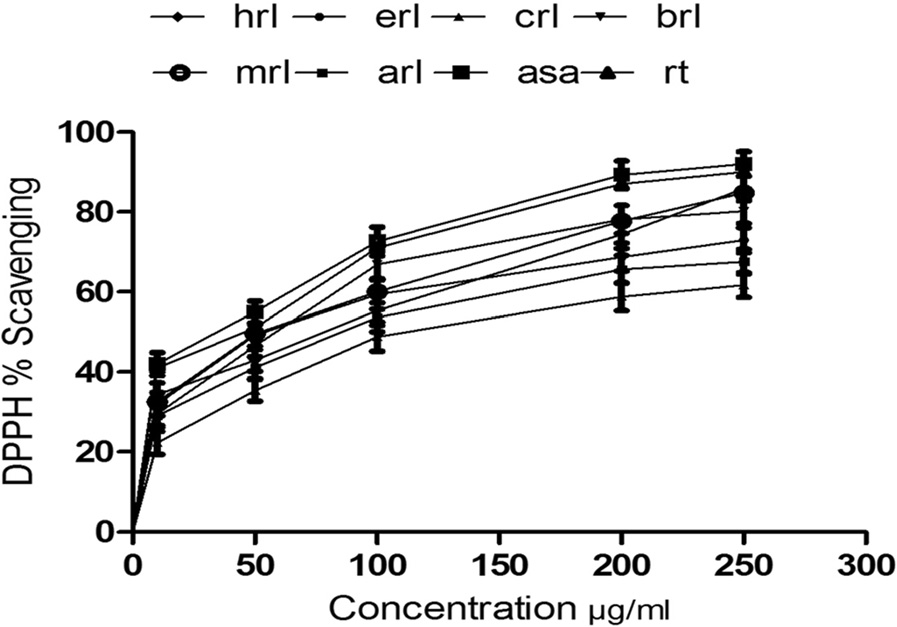
**DPPH radical scavenging activity of different fractions of *Maytenus royleanus *leaves at different concentrations.** Each value represents a mean ± SD (n = 3): Hrl., n-hexane fraction; erl, ethyl acetate fraction; crl, chloroform fraction; brl, butanol fraction; mrl, methanol fraction; arl, aqueous fraction; asa, ascorbic acid; rt, rutin.

DPPH radical scavenging activity of the *M. royleanus* is shown in Figure 1. In this study the methanol extract and various fractions dose dependently scavenged the DPPH radicals. IC_50_ values of DPPH radical scavenging ability of extract and its derived fractions can be ranked as ethyl acetate < methanol < *n*-butanol < *n*-hexane < residual aqueous < chloroform fraction. IC_50_ value determined for ethyl acetate fraction, methanol extract and *n*-butanol were nonsignificantly different from each other (Table 4). However, the scavenging activities of all fractions were less (P < 0.05) than those of standard compounds such as ascorbic acid and rutin.

**Table 4:** **Antioxidant effects of extract and its fractions of *M. royleanus *leaves**

**Extract/fraction**		**IC**_**50 **_**values**		
	**Scavanging ability on DPPH**	**Scavanging ability on**	**Phospho molybdate**	**Inhibition of lipid**
	**radicals**	**superoxide**	**Assay**	**peroxidation**
Methanol extract	55.02 ± 0.5^b^	69.80 ± 2.6^c^	46.10 ± 35^b^	79.12 ± 0.5^b^
*n*-hexane fraction	78.10 ± 1.2^c^	79.21 ± 2.8^d^	40.01 ± 0.7^a^	82.1 ± 1.8^c^
Chloroform fraction	109.8 ± 3.2^e^	250.10 ± 0.5^f^	90.22 ± 1.2^d^	130.21 ± 1.4^d^
Ethyl acetate fraction	55.01 ± 2.3^b^	47.01 ± 1.3^b^	39.02 ± 0.5^a^	76.32 ± 0.3^b^
*n*-butanol fraction	58.01 ± 0.7^b^	68.01 ± 4.8^c^	57.12 ± 0.6^c^	238.08 ± 0.5^e^
Residual aqueous fraction	87.21 ± 1.4^d^	104.07 ± 2.1^e^	>250^e^	80.01 ± 1.4^c^
Ascorbic acid	34.10 ± 0.8^a^	34.00 ± 0.6^a^	38.40 ± 2.4^a^	35.9 ± 0.7^a^
Rutin	44.70 ± 0.7^a^	-	-	-

Each value is represented as mean ± SD (n = 3). Values in the same column followed by a different letter (^a-f^) are significantly different (p < 0.05). -, not determined.

#### Superoxide radical scavenging activity

**Figure 2:**
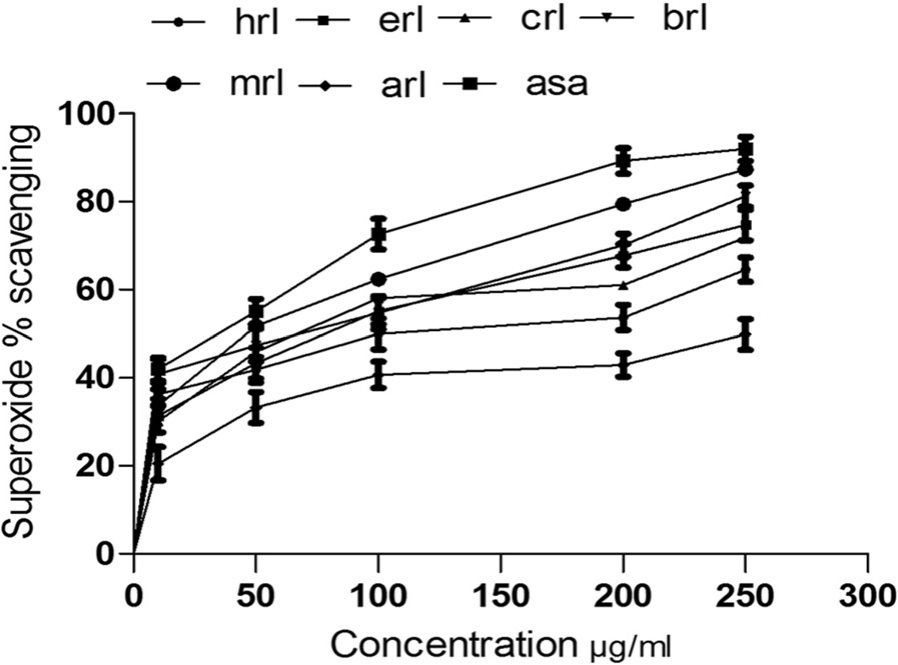
**Superoxide radical scavenging activity of different fractions of *Maytenus royleanus *leaves at different concentrations.** Each value represents a mean ± SD (n = 3): Hrl., n-hexane fraction; erl, ethyl acetate fraction; crl, chloroform fraction; brl, butanol fraction; mrl, methanol fraction; arl, aqueous fraction; asa, ascorbic acid; rt, rutin.

Crude extract and its derived fractions showed a scavenging activity on superoxide radicals in a concentration dependent manner (Figure 2). IC_50_ values for superoxide scavenging activities were in order of ethyl acetate < *n*-butanol < methanol < *n*-hexane < residual aqueous < chloroform fraction (Table 3). Ethyl acetate fraction exhibited the highest scavenging ability with IC_50_ value of 47.0 ± 1.3 μg/ml followed by *n*-butanol fraction (IC_50_ 68.0 ± 2.6 μg/ml) as compared to that of ascorbic acid (IC_50_ 34.0 ± 0.6 μg/ml).

#### Phosphomolybdate assay (Total antioxidant capacity)

The phosphomolybdate assay has been commonly used to determine the total antioxidant capacity of samples [40]. In this experiment Mo (VI) was reduced to Mo (V) by antioxidant potential of the extract and different fractions in a concentration dependent manner. The antioxidant capacity of the extract and different fractions was in the order of ethyl acetate > *n*-hexane > methanol > *n*-butanol > chloroform > residual aqueous fraction. The IC_50_ values for the ethyl acetate and *n*-hexane fractions were 39.0 ± 0.5 μg/ml and 40.0 ± 0.7 μg/ml, respectively, and were statistically (P > 0.05) similar to the total antioxidant capacity of ascorbic acid (Table 4).

### Reducing assays

#### Hydroxyl radical scavenging

The hydroxyl radical scavenging ability of different fractions can be ranked as ethyl acetate > *n*-hexane > methanol > *n*-butanol > chloroform > residual aqueous fraction. Results showed that hydroxyl radical scavenging capacity was increased by increasing the concentration of extract and all fractions at 25 - 250 μg/ml. The IC_50_ values of scavenging hydroxyl radicals for ethyl acetate fraction was 60.0 ± 3.2 μg/ml while for residual aqueous fraction it was > 250 μg/ml (Table 5).

**Table 5:** **Reducing power effects of extract and its fractions of *M. royleanus *leaves**

**Extract/fraction**		** IC**_**50 **_**values**	
	**Scavenging ability on**	**Scavenging ability on**	**Scavenging ability on**
	**hydroxyl radicals**	**hydrogen peroxide**	**ABTS radicals**
Methanol extract	137.02 ± 1.4^d^	70.05 ± 1.9^b^	211.10 ± 4.9^f^
*n*-hexane fraction	98.10 ± 0.5^c^	72.02 ± 0.7^b^	>250^g^
Chloroform fraction	>250^f^	>250^e^	65.01 ± 0.7^c^
Ethyl acetate fraction	60.01 ± 3.2^b^	89.00 ± 0.1^c^	78.00 ± 1.2^d^
*n*-butanol fraction	250.08 ± 1.3^e^	119.00 ± 0.2^d^	53.22 ± 2.2^b^
Residual aqueous fraction	> > 250^g^	123.02 ± 1.3^d^	116.11 ± 0.8^e^
Ascorbic acid	34.10 ± 1.1^a^	38.40 ± 0.7^a^	35.10 ± 1.5^a^
Rutin	-	45.01 ± 1.5^a^	-

Each value is represented as mean ± SD (n = 3). Values in the same column followed by a different letter (^a-g^) are significantly different (p < 0.05). -, not determined.

#### Hydrogen peroxide scavenging

Hydrogen peroxide scavenging activity determined in this study for the extract and different fractions of *M. royleanus*; were capable of scavenging hydrogen peroxide in a concentration dependent manner (25 - 250 μg/ml). As compared for IC_50_ values, the hydrogen peroxide scavenging activities of methanol (70.0 ± 1.9 μg/ml) and *n*-hexane fraction (72.0 ± 0.7 μg/ml) were more (P < 0.05) effective than that of chloroform (> 250 μg/ml) fraction (Table 5). The potential of all the fractions to scavenge hydrogen peroxide as shown by IC_50_ values were significantly different (P < 0.05) from the IC_50_ values obtained for standard compounds (ascorbic acid and rutin). The scavenging abilities on hydrogen peroxide were in order of methanol > *n*-hexane > ethyl acetate > *n*-butanol > residual aqueous > chloroform fraction.

#### ABTS radical scavenging

ABTS radical scavenging ability of the extract and different fractions can be ranked as *n*-butanol > chloroform > ethyl acetate > residual aqueous > methanol > *n*-hexane fraction. The scavenging ability of the extract and fractions for ABTS radicals was related with concentration of the tested samples. The *n*-butanol and chloroform fraction exhibited the highest scavenging potential when reacted with ABTS radicals as compared to the methanol and *n*-hexane fraction (Table 5). IC_50_ values of the extract and all fractions obtained were significantly (P > 0.05) higher than that of the standard compounds.

#### Reducing power assay

In reducing power assay, the reduction of ferric cyanide complex to the ferrous form by donating an electron indicates the presence of reductants in the testing samples. It was observed that the reducing power of the extract and different fractions increased in a concentration dependent manner. The reducing power of the extract and various fractions was in an order of *n*-butanol > methanol > chloroform > *n*-hexane > ethyl acetate and residual aqueous fraction. The *n*-butanol and methanol fraction exhibited a good reducing power of 1.45 ± 0.02 μg/ml and 1.24 ± 0.04 μg/ml at 250 μg/ml (Table 6).

**Table 6:** **Reduction of Fe**^**3+ **^**to Fe**^**2+ **^**of extract and different fractions of *M. royleanus *leaves**

**Plant extracts**	**Reducing power (700 nm,250 μg/ml)**
Methanol extract	1.55 ± 0.04^c^
*n*-hexane fraction	1.47 ± 0.01^c^
Chloroform fraction	0.95 ± 0.01^c^
Ethyl acetate fraction	1.50 ± 0.03^d^
*n*-butanol fraction	1.64 ± 0.02^b^
Residual aqueous fraction	1.03 ± 0.11^e^
Ascorbic acid	2.03 ± 0.04^a^

Each value is represented as mean ± SD (n = 3). Values in the same column followed by a different letter (^a-e^) are significantly different (P < 0.05).

### Anti-lipid peroxidation assay

On the basis of IC_50_ values pattern of anti-lipid peroxidation of the extract and various fractions can be arranged as ethyl acetate > methanol > residual aqueous > *n*-hexane > chloroform > *n*-butanol fraction. The results showed that approximately all the tested samples, dose dependently, inhibited lipid peroxidation. However, ethyl acetate fraction and methanol extract showed the highest scavenging potential while chloroform and *n*-butanol fraction showed the least anti-lipid peroxidation activity (Table 4). The IC_50_ values obtained for the extract and all fractions were significantly (P < 0.05) higher from the IC_50_ value obtained for ascorbic acid.

### Anti-hemotylic activity

Hemolytic activity of methanol extract of *M. royleanus* leaves and its various fractions was screened against normal human erythrocytes. Extract and different fractions exhibited differential pattern hemolytic effect towards human erythrocytes. Result indicated that the ethyl acetate fraction exhibited minimum hemolytic activity, where as aqueous fraction showed the highest hemolytic activity. Lysis of erythrocytes was found to be increased with an increase of extract or fraction concentration (Table 7).

**Table 7:** **Anti-hemolytic activity of extract and various fractions of *M. royleanus *leaves**

**Extract**	**Positive control **^**a**^	**Negative control **^**b**^	**Optical density **^**c**^				
			**Concentration μg/ml**				
			**10**	**50**	**100**	**200**	**250**
	0.41 ± 0.01	1.07 ± 0.02					
Methanol extract			1.03 ± 0.02 ^a^	0.94 ± 0.02 ^b^	0.88 ± 0.04 ^b^	0.84 ± 0.02 ^b^	0.78 ± 0.03 ^b^
	0.41 ± 0.01	1.07 ± 0.02					
*n*-hexane fraction			1.01 ± 0.03 ^a^	0.98 ± 0.03 ^a^	0.87 ± 0.02 ^b^	0.82 ± 0.04 ^b^	0.76 ± 0.04 ^b^
	0.41 ± 0.01	1.07 ± 0.02					
Chloroform fraction			0.94 ± 0.04 ^b^	0.76 ± 0.03 ^c^	0.74 ± 0.03 ^c^	0.68 ± 0.03 ^d^	0.64 ± 0.03 ^c^
	0.41 ± 0.01	1.07 ± 0.02					
Ethyl acetate fraction			1.03 ± 0.02 ^a^	0.98 ± 0.04 ^a^	0.96 ± 0.04 ^a^	0.92 ± 0.04 ^a^	0.85 ± 0.05 ^a^
	0.41 ± 0.01	1.07 ± 0.02					
*n*-butanol fraction			0.87 ± 0.03^c^	0.74 ± 0.04 ^c^	0.70 ± 0.04 ^c^	0.65 ± 0.04 ^c^	0.58 ± 0.04 ^d^
	0.41 ± 0.01	1.07 ± 0.02					
Residual aqueous fraction			0.81 ± 0.02 ^d^	0.76 ± 0.02 ^c^	0.71 ± 0.02 ^c^	0.64 ± 0.03 ^c^	0.54 ± 0.03 ^e^

Each value is represented as mean ± SE (n = 3). Values in the same column followed by a different letter (^a-e^) are significantly different (p < 0.05).

^a^Water replaced extract/fractions to serve as a positive control since this treatment results in 100% hemolysis.

^b^ Phosphate buffered saline replaced extract/fraction to serve as a negative control since this treatment results in 0% hemolysis.

^c^ Lower values are associated with increased cell lysis.

### Correlation between IC_50_ values with phenolic and flavonoid content

In this study association of total phenolic and flavonoid content with IC_50_ values of different *in vitro* antioxidant assays was estimated (Table 8). A significant correlation of IC_50_ values of DPPH, superoxide, hydrogen peroxide and anti-lipid peroxidation was established with total phenolic and flavonoid contents. However, a nonsignificant correlation was found between IC_50_ values of phosphomolybdate assay, ABTS and hydroxyl radical and total phenolic and flavonoid contents.

**Table 8:** **Correlation of total phenolics and flavonoids content with antioxidant assays of the extract and fractions of *M. royleanus *leaves**

** Assays**	**Correlation R**^**2**^	
	**Phenolics**	**Flavonoids**
IC_50_ of DPPH radical scavenging potential	0.9256^b^	0.9812^b^
IC_50_ of superoxide radical scavenging potential	0.7379^a^	0.7905^a^
IC_50_ of total antioxidant capacity	0.2568	0.1892
IC_50_ of hydroxyl radical scavenging potential	0.3843	0.3181
IC_50_ of hydrogen peroxide radical scavenging potential	0.7240^a^	0.6718^a^
IC_50_ of ABTS radical scavenging potential	0.1285	0.0354
IC_50_ of inhibition of lipid peroxidation	0.6542^a^	0.6587^a^

Methanol extract and its fractions of *M. royleanus* were used in correlation.

^a,b^ indicate significance at P < 0.05 & P < 0.01 respectively.

## Discussion

Phytochemical screening provides basic information about medicinal importance of a plant extract. In this study evaluation for qualitative and quantitative estimation of the chemical constituents of *M. royleanus* extracts showed the presence of various secondary metabolites. Phytochemical analysis of methanol extract of *M. royleanus* leaves revealed the presence of bioactive constituents such as alkaloids, anthraquinones, cardiac glycosides, coumarins, flavonoids, saponins, phlobatannins, tannins and terpenoids. These constituents were separated away on the basis of polarity of different solvents in to different fractions. Alkaloids and cardiac glycosides were absent in the chloroform fraction whereas flavonoids were not detected in the residual aqueous fraction. Ethyl acetate fraction was comprised of all the constituents except the anthraquinones. The biochemical investigation reports indicated the same composition of phytochemicals for the crude methanol extract of different plants [10,11,39].

**Figure 3:**
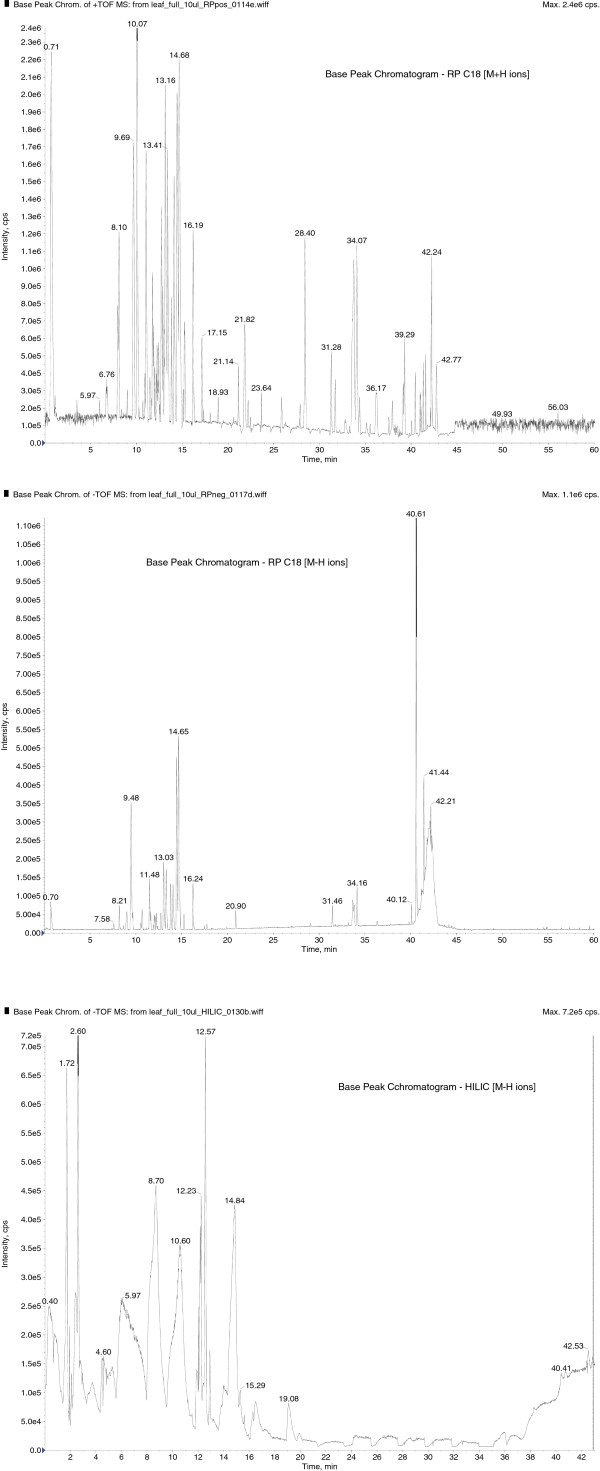
**LC-MS of methanol extract of *M. royleanus*, 1, Base peak chromatogram – RP C18 (M + H ions) 300 features. 2**, Base peak chromatogram – RP C18 (M-H ions) 59 features. **3**, Base peak chromatogram – HILIC (M-H Ions) 164 features.

The methanol extract of *M. royleanus* leaves were used to analyze the chemical composition by LC-MS. This technique was used to get a fingerprint of phytochemical composition of plant extract. As shown by the different compositions of the base peaks in the chromatograms, it is a complex mixture of different compounds such as phytoestrogens which are often found to be conjugated to one or more carbohydrate moieties and other polar groups (Figure 3). Further work is in progress to identify and isolate some of these compounds by elemental analysis and other analytical methods such as NMR spectroscopy or MS measurements.

Different species of *Maytenus* genus are used in traditional medicine for treatment of cancer [41] and for treatment of gastrointestinal diseases [42]. The biological activities of *Maytenus* species are considered to be due to the presence of different classes of secondary metabolites such as phenolic glucosides [43] flavonoids [44] and triterpenes. Pharmacological potential of *Maytenus* genus has been largely associated to the presence of triterpenes such as 3, 15-dioxo-21α-hydroxyfriedelane that was isolated from *Maytenus robusta*; showed antiulcerogenic activity [45]. Maytenfolic acid was isolated from *Maytenus herterophylla* and inhibited the growth of *Candida albicans*[46].

Quantitative determination of total flavonoid and total phenolic contents indicates that methanol extract possesses the highest concentration of total flavonoid and phenolic content. Similar findings have been reported where total phenolic and flavonoid contents are found in greater quantity in different plants [10,11,40]. The chemical complexity of extract/fraction, often a mixture of varied compounds; having different functional groups, polarity and chemical behavior, could lead to diverse results, depending on the test employed. The solvents such as methanol, ethyl acetate and *n*-butanol are found superior in concentrating the phenolics from *M. royleanus*. It has been reported that rich flavonoid and phenolic plants could be a vital source of therapeutic potential against the oxidative damages [12,13,47].

The scavenging effect of the extract and various fractions of *M. royleanus* leaves against DPPH radicals varied a great deal indicating that scavenging activities are related to the electron transfer/donating ability. The extract and all the fractions exhibited inferior IC_50_ values for DPPH radical scavenging activity as compared to the ascorbic acid. However, methanol extract and ethyl acetate fraction exhibit comparatively high antioxidant capacity as against the other fractions; suggesting the presence of free radical inhibitors acting possibly as primary antioxidants. Some of the pharmacological effects could be attributed due to these compounds.

Superoxide radical scavenging activity of different fractions was increased dose dependently in this study. Low level of IC_50_ values suggested that the chemical constituents found in methanol extract and its fractions are potent scavengers of superoxide radical at low concentration. Superoxide is one of the precursors of hydroxyl or singlet oxygen species, and also can propagate other free radicals, induces intricate process of lipid peroxidation resulting in membranous system damage and cellular injuries. The strong electron quenching activity of the extract and fractions could be due to the antioxidant compounds such as flavonoids and phenolic contents that enable to scavenge the oxidation of biological macromolecules [10,11].

Ethyl acetate fraction of *M. royleanus* leaves depicted the highest level of total antioxidant capacity in the present investigation. It is suggested that many flavonoid and polyphenolics found in medicinal plants contribute significantly to the phosphomolybdate scavenging activity.

Antioxidant potential of extract or fraction is directly related to the scavenging of hydroxyl radical and consequently the inhibition of lipid peroxidation [47]. Generation of lipid hydroperoxides can cause damage to every molecule of the biological system and have the capacity to bind with DNA causing strand breaks, carcinogenesis and mutation [48]. The erythrocytes intrinsically are more prone to peroxidation due to the heavy accumulation of polyunsaturated fatty acids and haemoglobin. During respiration erythrocytes are continuously exposed to high tension of oxygen, which can induce oxidative damage [49]. Further, exposure of erythrocytes to toxicants leads to the generation of free radicals resulting in potential damages to the membranes and consequently hemolysis [50]. Extract and fractions used in this experiment exhibit the scavenging of hydroxyl radicals suggesting the presence of primary antioxidants which possess anti hemolytic as well as anti-lipid peroxidation potential.

Hydrogen peroxide itself is not very toxic to the cellular system; sometimes it becomes injurious as it is directly involved in the generation of hydroxyl radicals, act as free reactive oxygen species [11]. In this study methanol extract and its derived fractions such as *n*-hexane and ethyl acetate have moderate potential to scavenge the hydrogen peroxide potential hazard indicating the antioxidant capacity of the plant.

ABTS radical scavenging ability of the extract and different fractions exhibited a dose dependent response. Recent studies have shown that polyphenolics such as catechin, rutin and their derivatives more effectively scavenge the ABTS radicals [51]. Highest ABTS radical scavenging potential was shown by the *n*-butanol fraction indicating more accumulation of polyphenolics in this fraction.

In the reducing power assay the antioxidants donate an electron to stabilize the radicals and also break the free radical chain reaction [52]. The ability of fractions to exhibit the reducing power in this investigation may be related with the presence of antioxidant phytochemicals.

*Maytenus royleanus* possesses diverse antioxidant compounds as depicted by the antioxidant activities of different fractions. A strong correlation of IC_50_ values of antioxidant assays; DPPH, superoxide and hydrogen peroxide; with phenolic and flavonoids content of *M. royleanus* was exhibited in this study. However, nonsignificant correlation was found for IC_50_ values of total antioxidant capacity, ABTS and hydroxyl radical scavenging potential with flavonoid and phenolic contents. These results indicate that flavonoid and phenolics can be the major contributors for the antioxidant activity observed for the *M. royleanus* leaves extract and its fractions. These fractions may be exploited for the antioxidant potential of this plant.

## Conclusions

Strong antioxidant capacity of the extract and its derived fractions for different *in vitro* antioxidant assays may be related with the antioxidant constituents such as flavonoid, phenolic, tannins and terpenoids in this plant. These results suggested the use of ethyl acetate fraction as primary antioxidant therapeutic source.

## Competing interest

The authors declare that they have no competing interests.

## Authors’ contributions

MS has made substantial contribution to acquisition of data, analysis, drafting of the manuscript. MRK has made substantial contribution to conception and design, interpretation of data, drafting and revising the manuscript for intellectual content. NS participated in the design and collection of data and analysis. All authors read and approved the final manuscript.

## Pre-publication history

The pre-publication history for this paper can be accessed here:

http://www.biomedcentral.com/1472-6882/13/143/prepub

## References

[B1] Ivanova D Gerova D Chervenkov T Yankova T Polyphenols and antioxidant capacity of Bulgarian medicinal plants J Ethnopharmacol 2005 97 145 150 10.1016/j.jep.2004.10.023 15588663

[B2] Gulcin I Antioxidant activity of food constituents-An overview Arch Toxicol 2012 86 3 339 345 10.1007/s00204-012-0813-7 22102161

[B3] Gulcin I Beydemir S Phenolic compounds as antioxidants: Carbonic anhydrase isoenzymes inhibitors Mini Rev Med Chem 2013 13 3 408 430 23190033 10.2174/138955713804999874

[B4] Balasundram N Sundram K Sammar S Phenolic compounds in plants and agri-industrial by-products: Antioxidant activity, occurrence, and potential uses Food Chem 2006 68 191 203

[B5] Aruoma IO Cuppette SL Antioxidant methodology in vivo and in vitro concept 1997 Illinois: AOAS press

[B6] Marchand LL Cancer preventive affects of flavonoids: a review Biomed Pharmacother 2002 56 296 301 10.1016/S0753-3322(02)00186-5 12224601

[B7] Khan MR Rizvi W Khan GN Khan RA Shaheen S Carbon tetrachloride-induced nephrotoxicity in rats: protective role of *Digera muricata* J Ethnopharmacol 2009 122 91 99 10.1016/j.jep.2008.12.006 19118616

[B8] Khan RA Khan MR Sahreen S Evaluation of *Launea procumbens* use in renal disorders: a rat model J Ethnopharmacol 2010 128 452 461 10.1016/j.jep.2010.01.026 20096342

[B9] Khan MR Haroon J Khan RA Bokhari J Rashid U Prevention of KBrO_3_-induced cardiotoxicity by *Sonchus asper* in rat J Med Plants Res 2011 5 2514 2520

[B10] Sahreen S Khan MR Khan RA Evaluation of antioxidant activities of various solvent extracts of *Carissa opaca* fruits Food Chem 2010 122 1205 1211 10.1016/j.foodchem.2010.03.120

[B11] Sahreen S Khan MR Khan RA Phenolic compounds and antioxidant activities of *Rumex hastatus* D. Don. Leaves J Med Plants Res 2011 5 2755 2765

[B12] Bursal E Koksal E Gulcin I Bilsel G Goren AC Antioxidant activity and polyphenol content of cherry stem (*Cerasus avium* L.) determined by LC-MS/MS Food Res Int 2013 51 1 66 74 10.1016/j.foodres.2012.11.022

[B13] Gulcin I Topal F Cakmakc R Bilsel M Goren AC Erdogan U Pomological features, nutritional quality, polyphenol content analysis and antioxidant properties of domesticated and three wild ecotype forms of raspberries (*Rubus idaeus* L.) J Food Sci 2011 76 4 C585 C593 10.1111/j.1750-3841.2011.02142.x 22417339

[B14] Ellof JN Which extract should be used for the screening and isolation of antimicrobial components from plant J Ethnopharmacol 1998 60 1 8 10.1016/S0378-8741(97)00123-2 9533426

[B15] Middleton E Jr Kandaswami C Theoharides TC The effects of plant flavonoids on mammalian cells: implications for inflammation, heart disease and cancer Pharmacol Rev 2000 52 673 751 11121513

[B16] Velioglu YS Mazza G Gao L Oomah BD Antioxidant activity and total phenolics in selected fruits, vegetables and grain products J Agri Food Chem 1998 46 4113 4117 10.1021/jf9801973

[B17] Gulcin I Kufrevioglu OI Oktay M Buyukokuroglu ME Antioxidant, antimicrobial, antiulcer and analgesic activities of nettle (*Urtica dioica* L.) J Ethnopharmacol 2004 90 205 215 10.1016/j.jep.2003.09.028 15013182

[B18] Gulcin I Tel AZ Kirecci E Antioxidant, antimicrobial, antifungal and antiradical activities of *Cyclotrichium niveum* (Boiss.) Manden and Scheng Int J Food Prop 2008 11 2 450 471 10.1080/10942910701567364

[B19] Nasir ASI Flora of West Pakistan. No. 100 1977 Karachi: Feroz sons 1

[B20] Samin J Khan MA Din SU Murad W Hussain M Ghani A Herbal remedies used for gastrointestinal disorders in Kaghan Valley, NWFP, Pakistan Pak J Weed Sci Res 2008 14 169 200

[B21] Kil HY Seong ES Ghimire BK Chung IM Kwon SS Goh EJ Hoe K Kim MJ Lim JD Lee D Yu CY Antioxidant and antimicrobial activities of crude *Sorghum* extract Food Chem 2009 115 1234 1239 10.1016/j.foodchem.2009.01.032

[B22] Harborne JB Phytochemical methods 1973 London: London Chapman and Hall, Ltd 49 188

[B23] Trease GE Evans WC Pharmacognosy 1989 London: Brailliar Tiridel Can Macmillian Publishers 11

[B24] Sofowora AE Medicinal plants and traditional medicine in Africa, Volume 2 1993 Ibadan, Nigeria: Spectrum books Ltd 289

[B25] Kim DO Jeong SW Lee CY Antioxidant capacity of phenolic phytochemicals from various cultivars of plums Food Chem 2003 81 321 326 10.1016/S0308-8146(02)00423-5

[B26] Brand-Williams W Cuvelier ME Berset C Use of free radical method to evaluate antioxidant activity Lebensmittel-Wissenschaftund-Technol 1995 28 25 30

[B27] Yong SP Soon TJ Seong GK Buk GH Patricia AA Fernando T Antioxidant and proteins in ethylene-treated kiwifruits Food Chem 2008 107 640 648 10.1016/j.foodchem.2007.08.070

[B28] Gulcin I Antioxidant properties of resveratrol: A structure-activity insight Innov Food Sci Emerg Technol 2010 11 210 218 10.1016/j.ifset.2009.07.002

[B29] Gulcin I Antioxidant activity of L-Adrenaline: An activity-structure insight Chem Biol Interact 2009 179 2–3 71 80 18929549 10.1016/j.cbi.2008.09.023

[B30] Beauchamp C Fridovich I Superoxide dismutase: improved assays and an assay applicable to acrylamide gels Anal Biochem 1971 1971 44 276 277 4943714 10.1016/0003-2697(71)90370-8

[B31] Umamaheswari M Chatterjee TK In vitro antioxidant activities of the fractions of *Coccinnia grandis* L. leaf extract Afr J Trad Compl Altern Med 2008 5 61 73 PMC2816591 20162057

[B32] Halliwell B Gutteridge JMC Formation of thiobarbituric acid reactive substances from deoxyribose in the presence of iron salts: the role of superoxide and hydroxyl radicals FEBS Lett 1981 128 347 352 10.1016/0014-5793(81)80114-7 6266877

[B33] Ruch RJ Cheng SJ Klaunig JE Prevention of cytotoxicity and inhibition of intercellular communication by antioxidant catechins isolated from Chinese green tea Carcinogen 1989 10 1003 1008 10.1093/carcin/10.6.1003 2470525

[B34] Gulcin I Buyukokuroglu ME Kufrevioglu OI Metal chelating and hydrogen peroxide scavenging effects of melatonin J Pineal Res 2003 34 278 281 10.1034/j.1600-079X.2003.00042.x 12662350

[B35] Oktay M Gulcin I Kufrevioglu OI Determination of in vitro antioxidant activity of fennel (*Foeniculum vulgare*) seed extracts Lebensm Wiss Technol 2003 36 2 263 271 10.1016/S0023-6438(02)00226-8

[B36] Re R Pellegrini N Proteggente A Pannala A Yang M Rice-Evans C Antioxidant activity applying an improved ABTS radical cation decolorisation assay Free Radical Bio Med 1999 26 1231 1237 10.1016/S0891-5849(98)00315-3 10381194

[B37] Oyaizu M Antioxidant activity of browning products of glucosamine fractionated by organic solvent and thin layer chromatography Nippon Shokulin Kogyo Gakkaishi 1986 35 771 775

[B38] Ohkawa H Ohishi N Yagi K Assay for lipid peroxides in animal tissues by thiobarbituric acid reaction Anal Biochem 1979 95 351 358 10.1016/0003-2697(79)90738-3 36810

[B39] Yang ZG Sun HX Fang WH Haemolytic activities and adjuvant effect of *Astragalus* membranaceus saponins (AMS) on the immune responses to ovalbumin in mice Vaccine 2005 23 5196 5203 10.1016/j.vaccine.2005.06.016 16043270

[B40] Prieto P Pineda M Aguliar M Spectrophotometric quantitation of antioxidant capacity through the formation of Phosphomolybdenum complex: Specific application to the determination of vitamin E Anal Biochem 1999 269 337 341 10.1006/abio.1999.4019 10222007

[B41] Khan RA Khan MR Sahreen S Ahmed M Evaluation of phenolic contents and antioxidant activity of various solvent extracts of *Sonchus asper* (L.) Hill Chem Cent J 2012 6 12 10.1186/1752-153X-6-12 22305477 PMC3292812

[B42] Schaneberg BT Green DK Sneden AT Dihydroagarofuran sesquiterpene alkaloids from *Maytenus putterlickoides* J Nat Prod 2001 64 624 626 10.1021/np010041o 11374957

[B43] Cordeiro PJM Vilegas JHY Lancas FM HRGC-MS analysis of terpenoids from *Maytenus ilicifolia* and *Maytenus aquifolium* (“Espinheira Santa”) J Braz Chem Soc 1999 10 523 526 10.1590/S0103-50531999000600017

[B44] Sannomiya M Vilegas W Ratrelli L Pizza C A flavonoids glycoside from *Maytenus aquifolium* Phytochemistry 1998 49 237 239 10.1016/S0031-9422(97)00842-X

[B45] Da Silva MS De Sousa DP Medeiros VM Folly MAB Tavares JF Barbosa-Filho JM Alkaloid, flavonoids and pentacyclic triterpenoids of *Maytenus obtusifolia* Mart Biochem Syst Ecol 2008 36 500 503 10.1016/j.bse.2008.01.006

[B46] Andrade SF da Silva Filho AA de O Resende D Silva ML Cunha WR Nanayakkara NP Bastos JK Antileishmanial, antimalarial and antimicrobial activities of the extract and isolated compounds from *Austroplenckia populnea* (Celastraceae) Z Naturforsch C 2008 63 497 502 18810991 10.1515/znc-2008-7-805

[B47] Orabi KY Al-Qasoumi SI EI-Qlemy MM Mossa JS Muhammad I (Dihydroagarofuran alkaloid and triterpenes from *Maytenus heterophylla* and *Maytenus arbutifolia* Phytochemistry 2001 58 475 480 10.1016/S0031-9422(01)00277-1 11557080

[B48] Sharififar F Dehghan-nudeh GH Mirtajaldini M Major flavonoids with antioxidant activity from *Teucrium polium* L Food Chem 2009 112 885 888 10.1016/j.foodchem.2008.06.064

[B49] Babu BH Shylesh BS Padikkala J Antioxidant and hepatoprotective effect of *Alanthus icicifocus* Fitoterapia 2001 72 272 277 10.1016/S0367-326X(00)00300-2 11295303

[B50] Chakraborty D Verma RJ Ameliorative effect of *Emblica officinalis* aqueous extract against ochratoxin – induced lipid peroxidation in the kidney and liver of mice Int J Occup Med Environ Health 2010 23 1 11 10.2478/v10001-010-0009-4 20442064

[B51] Rahimtula AD Bereziat JC Bussacchini-Griot V Bartsch H Lipid peroxidation as a possible cause of ochratoxin A toxicity Biochem Pharmacol 1988 37 4469 4477 10.1016/0006-2952(88)90662-4 3202889

[B52] Niki E Antioxidants in relation to lipid peroxidation Chem Phys Lipids 1982 44 227 253 3311418 10.1016/0009-3084(87)90052-1

